# Developing and assessing the usability of a prescribing quality and safety dashboard in Irish general practice: protocol for a qualitative study

**DOI:** 10.12688/hrbopenres.14181.1

**Published:** 2025-06-18

**Authors:** Caroline McCarthy, Tom Fahey, Frank Moriarty, Michelle Flood, Eimear Loftus, Barbara Clyne

**Affiliations:** 1 Department of General Practice, Royal College of Surgeons in Ireland (RCSI) University of Medicine and Health Sciences, Dublin 2, Ireland; 2 School of Pharmacy and Biomolecular Sciences, (RCSI) University of Medicine and Health Sciences, Dublin 2, Ireland; 3 Department of Public Health & Epidemiology, RCSI, University of Medicine and Health Sciences, Dublin 2, Ireland

**Keywords:** interactive dashboard, prescribing safety, patient safety, medication optimisation, electronic healthcare records, drug utilisation

## Abstract

**Background:**

The third WHO Global patient safety challenge, “Medication Without Harm” aims to reduce severe avoidable medication related ham by 50% globally. One approach to reducing medication related harm is to develop interventions that improve prescribing. Audit and feedback is one such intervention that has been shown to have an effect on professional behaviour. With advancements in the data infrastructure of primary care it is now possible to harness routine prescribing data for ongoing and up-to-date comparative benchmarking. The aim of this study was to assess the usability and usefulness of a prescribing safety dashboard developed in Irish general practice.

**Methods:**

Practices utilising the data analytics platform, MedVault opted in to share anonymous prescription data to enable the development of the prescribing safety dashboard. Participants from these practices who had previously expressed an interest in taking part in this qualitative study will be formally invited to take part. Recruited prescribers will take part in an online interview with a think aloud process where they will share their screen as they navigate the dashboard and verbalise their thoughts. This will be followed by a semi-structured interview where their views on prescribing safety and receiving feedback on prescribing will be explored. For the think aloud process, screen recordings will be reviewed alongside the transcripts, and analysed using Nielsen’s five quality components of usability: learnability, efficiency, memorability, error recovery and satisfaction as a framework. An inductive thematic approach will be used to analyse GPs’ perspectives on prescribing safety and feedback.

**Discussion:**

This study will explore the usability and acceptability of a prescribing quality and safety dashboard. To design future interventions, policies, and quality improvement initiatives that make use of routine data, it is essential to understand how GPs engage with and use these tools. This understanding can help ensure such initiatives are developed in ways that maximise relevance, usability, and engagement.

## Introduction

Medication-related harm is a major contributor to preventable patient morbidity and healthcare burden ^1^, for example almost 9% of emergency department admissions in older people are due to preventable drug related problems ^2^. Estimates for the prevalence of sub-optimal prescribing are even higher ranging from 20% to 50% ^3^. Internationally, initiatives such as the WHO’s Global Patient Safety Challenge Medication Without Harm recognise this growing problem and aim to reduce avoidable medication-related harm.

Alongside direct medication-related harm, overprescribing is a growing concern and refers to the use of medicines that are ineffective, have an unfavourable risk - benefit ratio or do not align with patient preferences. Estimates suggest that up to 10% of medications are overprescribed ^4^. Overprescribing is a systemic problem influenced by multiple factors, including pharmaceutical marketing and a lack of transparency in regulation and access to trial data, cultural and societal expectations that position medicines as quick-fix solutions and prescriber attitudes and knowledge. Drug utilisation rates and geographic patterns of variation can help characterise and describe overprescribing ^5^.

One approach to address high-risk prescribing and overprescribing is to target prescriber behaviour through audit and feedback interventions. There is evidence that audit and feedback interventions have a small but significant effect on health professional behaviour, particularly when repeated and sustained exposure is used ^6^. This finding has subsequently been confirmed with suggestions that the current focus should be to systematically test various approaches to the design and development of audit and feedback ^7^. With advances in electronic prescribing and data infrastructure, it is now possible to provide real-time, comparative feedback to prescribers about their prescribing patterns relative to their peers ^8^. While comparative prescribing dashboards have the potential to influence behaviour, their effectiveness depends on how they are used and engaged with. Given that most prescribing occurs in primary care, targeting this population is likely to have the greatest potential for impact on overall prescribing quality and safety.

This study aims to assess how GPs in Ireland engage with and use a prescribing safety dashboard. This will inform the design and implementation of future real-time prescribing feedback interventions. The first objective is to explore how prescribers interact with, interpret, and use the dashboards using a think-aloud approach. The second objective is to assess the perceived usefulness and acceptability of prescribing feedback, both generally and in this specific format, through semi-structured interviews.

## Methods

**Figure 1.**
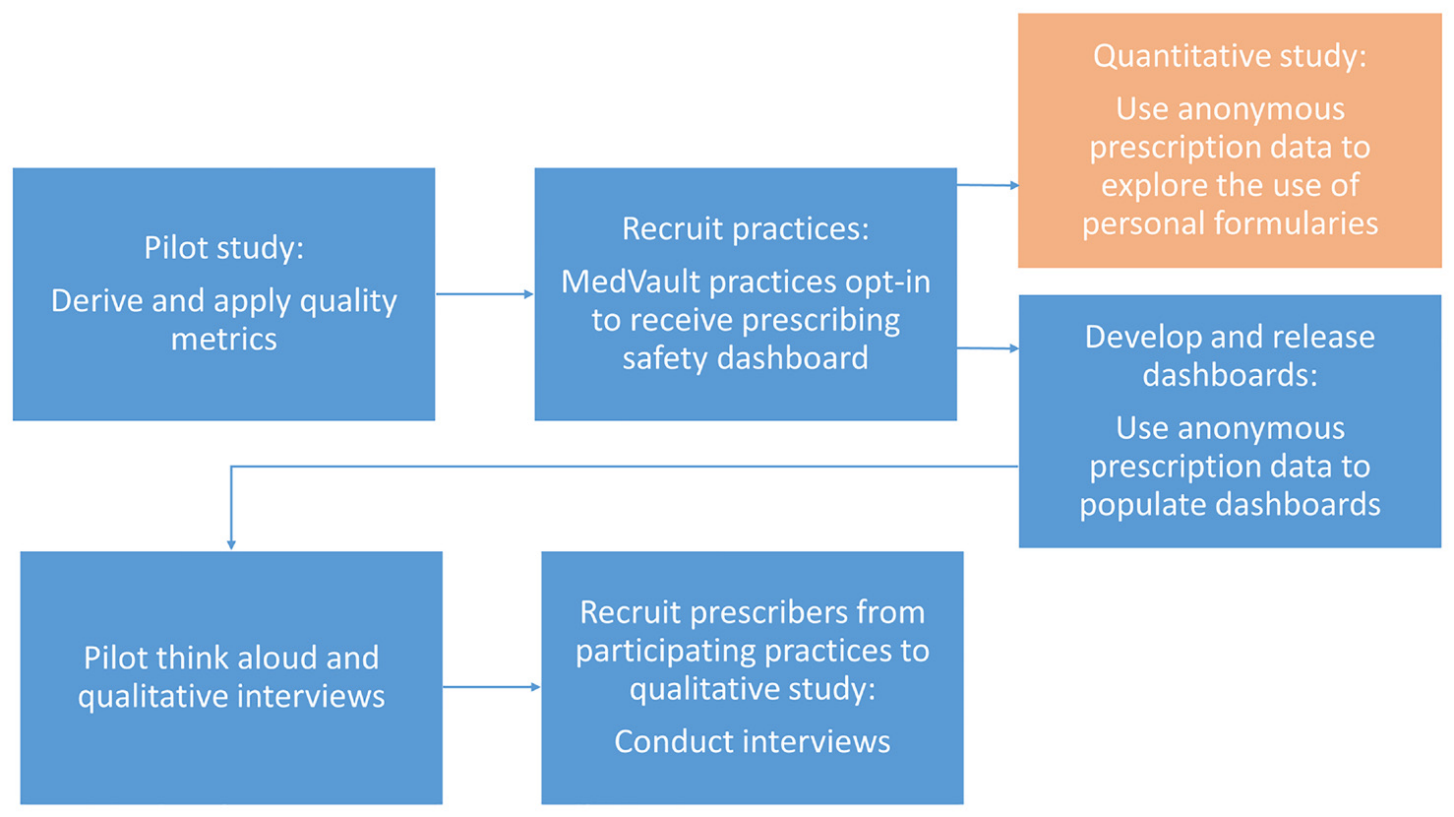
Project flow diagram.

In this qualitative study, a think-aloud exercise will be performed with prescribers to assess the usability of the interactive prescribing dashboards. Semi-structured online interviews will also be conducted to explore views on the acceptability and usefulness of the dashboards. This study adheres to the Declaration of Helsinki and ethical approval was received from the Irish College of General Practitioners Research Ethics Committee (ICGP_REC_2024_ 2502). All participants will give fully informed written consent. This qualitative study is part of a larger project which aims to explore the use of personal formularies in general practices, how this relates to the quality of prescribing, and how feedback using this information can be delivered to prescribers for comparative benchmarking, see Figure 1. The process of deriving and feeding back prescribing quality metrics was piloted in a previous study, where participating prescribers received a paper-based report comparing their performance to that of their peers ^9^.

### Setting

This study is set in general practice in the Republic of Ireland, where there is a mix of eligibility in terms of access to primary care and medicines. Individuals covered under the means tested General Medical Services (GMS) scheme, are eligible for free prescription medicines, with a small co-payment. In 2023 this represented 31% of the population. For those not covered by the GMS scheme there is a monthly threshold of €80 per household for the cost of prescribed medications, beyond which the state covers any further costs ^10^. Individuals covered by the GMS scheme, and approximately 10% of the population covered by the Doctor Visit scheme, have access to free GP visits and are registered with a single GP. However, the majority of patients (private patients) pay for GP visits and may not be registered with a single practice, making it challenging to calculate an accurate practice population denominator.

Within Europe, the UK and Nordic countries have led the way in advanced e-prescribing infrastructure with a central repository which can then be used for feedback ^11– 13^. In the UK there is routine national level prescribing surveillance where prescribing is actively monitored through structured frameworks, using prescribing data to provide feedback, financial incentives, and targeted interventions for GPs ^14^. Ireland relies more on voluntary adherence to Health Service Executive (HSE) guidance. Examples of such guidance include the Preferred Drugs initiative which provides recommendations on the use of preferred drugs within ten common drug classes that are selected based on effectiveness, cost effectiveness and practical considerations such as dosing regimens ^15^. Similarly, the HSE Antimicrobial Resistance and Infection Control team has developed guidance for community settings on “green” antibiotics, which are preferred to minimise the risk of anti-microbial resistance and adverse drug reactions ^16^. GPs currently receive feedback on benzodiazepine and antibiotic prescribing from the HSE based on dispensing data from the GMS scheme. However, this may not give the full picture compared to GP prescription data which includes both private prescriptions and medicines covered under drug re-imbursement schemes.

### Dashboard description

**Figure 2.**
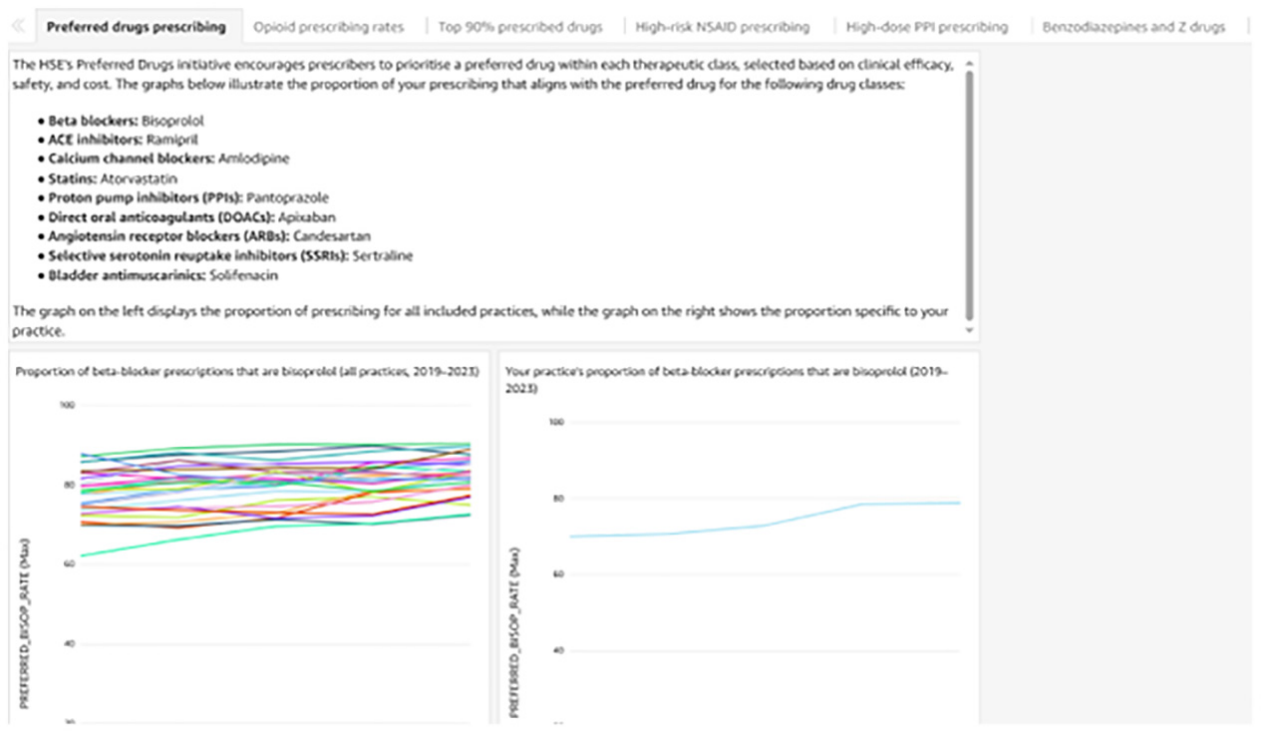
Sample page from prescribing quality and safety dashboard.

In collaboration with the data analytics platform, MedVault (a private company that supports general practices in using their clinical data to improve care and manage reimbursement claims), the lead author (CMC) has developed prescribing safety dashboards using Snowflake for data processing and Amazon QuickSight for visualisation. Practices will have ongoing access to their dashboards from February 2025 for six months. Each practice will be able to view their own prescribing data and compare it to aggregate data from other participating practices, see Figure 2. However, practices will remain anonymous to one another, MedVault and the research team.

The prescribing dashboard was designed to provide insights into prescribing patterns at the practice level. As practices have one unique login, it is not feasible to provide prescriber level feedback. The dashboards will initially display trend data over five years with comparative benchmarking, with a subsequent update in May 2025. Table 1 lists the included metrics with reasons for their inclusion.

**Table 1.** Included prescribing metrics.

Metric	Expressed as	Reason for inclusion
Antibiotic prescribing rate	Annual rate per 1,000 patients	National and international focus on reducing AMR ^16^ GPs have reived national comparative feedback since July 2018 but this is just for GMS patients ^19^
Antibiotic appropriateness	Annual proportion of all antibiotics that are green	National and international focus on reducing AMR ^16^ GPs have reived national comparative feedback since July 2018 but this is just for GMS patients ^19^
BZRA prescribing rate	Annual rate per 1,000 patients	Risk of dependence, harm and street diversion National strategies to address prescribing GPs have reived national comparative feedback since May 2017 but this is just for GMS patients ^20^
Potent opioid prescribing rate	Annual rate per 1,000 patients	Ineffective and dose dependent risk of harm when used for chronic pain Ongoing increasing rates of long term opioid prescriptions ^21^
High-dose PPI prescribing	Annual proportion of all PPI prescribing hat is high-dose	Potentially inappropriate high dose PPI prescribing is highly prevalent ^18^ GPs currently do receive feedback on PPI prescribing
High-risk NSAID prescribing: anticoagulant	Numerator is number of patients with > 1 anticoagulant prescriptions in that year Denominator is the number of these patients with at least one NSAID prescription that year	Risk of gastrointestinal bleeding ^22^ GPs currently do not receive any feedback on this metric
High-risk NSAID prescribing: ACEi/ARB AND diuretic	Numerator is number of patients with >1 prescription for both an ACEi/ARB and diuretic that year Denominator is the number of these patients with at least one NSAID prescription that year	Risk of acute kidney injury ^23^ GPs currently do not receive any feedback on this metric
Preferred drugs prescribing	For each of the ten drugs, expressed as a proportion of the total prescribing for that drug group in that year	HSE guidance on preferred drugs ^15^ Potential for cost savings if adhered to 24 Not currently monitored
Use of practice formulary	Drug Utilization 90% (DU90%) - Number of unique medicines accounting for 90% of practice prescribing for that year. Name and number of prescriptions for each medicine included in the DU90% segment ^25^	WHO Guide to Good Prescribing recommends use of personal formulary ^26^ Feasible to apply the DU90% indicator to anonymous prescription data ^9^ Use of the DU90% in another setting as a tool for feeding back data on prescribing quality acceptable and useful for GPs ^27^

*Abbreviations; AMR; antimicrobial resistance, BZRA; benzodiazepine and z-drug receptor agonists, GMS; general medical services, PPI; proton pump inhibitor, NSAID, non-steroidal anti-inflammatory drug, ACEi; angiotensin converting enzyme inhibitor, ARB; aldosterone receptor blocker, DU90%; drug utilisation 90%, WHO; World Health Organisation*

### Dashboard development

Data from participating practices (n=27) were scrubbed from practice servers by MedVault, see Table 2, and transformed within Snowflake, where key prescribing safety metrics were generated and structured for visualisation.

**Table 2.** Data points included in the analysis.

Variable	Description
Patient ID	Anonymous patient ID
Clinic ID	Anonymous practice ID
Prescriber ID	Anonymous prescriber ID
Prescription issue date	Date and time prescription was issued
Prescription type	GMS or private prescription
Drug name	Drug name as prescribed (either generic or brand) with strength e.g., Amlodipine 5mg tabs
Generic name	Generic drug name
ATC code	WHO ATC code
Current repeats	The number of times the prescription was issued (typically either 1 for acute items or 3 or 6 for repeat items)
Total quantity taken	The total number of that drug strength prescribed for that month (e.g., for Amlodipine 5mg this variable would be 30)
No of private patients in clinic	The number of registered patients with at least one encounter in the previous 3 years (calculated annually)
No of GMS patients in clinic	The number of registered GMS patients
Total number of patients	No of private patients + No of GMS patients
System	The practice management software used by the practice

**Figure 3.**
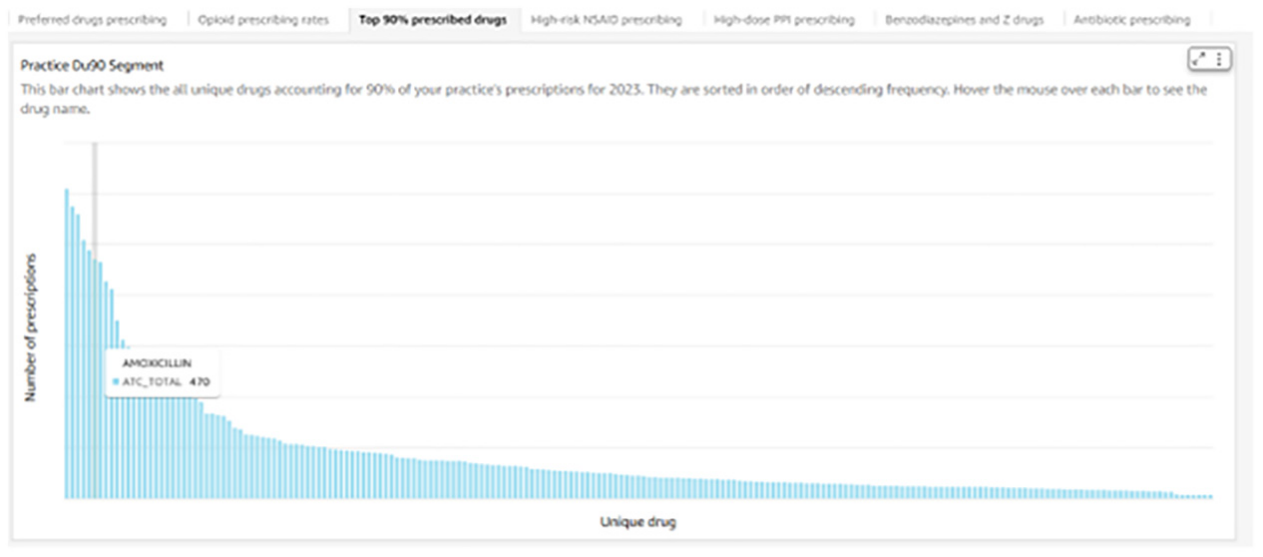
Practice DU90% segment.

The WHO Anatomical Therapeutic Chemical (ATC) classification system, which categorises medicines according to the organ or system they act on and their therapeutic, pharmacological and chemical characteristics ^17^, was used to identify drug groups and classes. There were errors in the WHO ATC code mapping process within practice systems so this was updated using a codebook developed for prior research that mapped branded and generic drug names to WHO ATC codes ^18^. Using the high-dose PPI prescribing metric as an example, all PPIs were identified using the ATC code beginning with A02BC. For metrics displayed as prescribing rates, rates were calculated annually using the total practice population, whereby numbers of private patients were calculated by counting individuals who had at least one encounter in the preceding three years. An encounter was defined as any recorded contact with the practice (including a prescription, telephone consultation or nurse consultation). High-dose PPI prescribing was the only metric that required dose information. The relevant dose was identified by searching the drug name variable for dose indicators corresponding to each PPI in the dataset (e.g., '40' for omeprazole, '30' for lansoprazole). For high-risk non-steroidal anti-inflammatory drug (NSAID) prescribing, the anonymous patient ID was used to identify instances where patients who had received more than one prescription for either an angiotensin-converting enzyme (ACE) inhibitor or angiotensin II receptor blocker and a diuretic had also received at least one prescription for a NSAID in the same year. Instances of patients who had received more than one prescription for an anticoagulant and at least one NSAID prescription in the same year were identified in a similar manner. For all comparative metrics, an identical dataset was cloned in Snowflake and imported into QuickSight with row-level permissions applied, ensuring that each practice could only view its own data. This graph was displayed alongside a graph showing data from all practices, allowing each practice to compare its performance with others. The DU90% indicator ( Table 1) was calculated on an annual basis for each practice. The total number of unique ATC coded items were counted by practice and by year and then sorted in descending order, so the cumulative proportion of prescribing for each drug could be calculated. Items whereby the cumulative proportion was <90% were identified as belonging in the DU90% segment for that practice in that year. A bar chart displaying all the drugs in descending order of quantity prescribed in each practice DU90% was developed in QuickSight, see Figure 3. This was the only metric that was not comparative. See extended data for the SQL code used to derive prescribing safety metrics.

### Study population

General practices in the Republic of Ireland that currently use the MedVault data analytics platform are eligible to participate. While the participating practices represent only a small proportion of practices nationally, they are broadly representative in terms of size and location and use two of the three predominant electronic health record systems in Ireland. MedVault practices who had previously consented to share anonymous prescription data (n=27) and receive interactive prescribing dashboards were asked to consent to be contacted about this qualitative study. Contact details for GPs interested in participating in this qualitative study were stored separately and not linked to prescribing data. For this study a purposive sample of approximately 12 prescribers from different practices will be recruited to ensure a range of perspectives and experiences. This sample size was chosen based on the concept of information power, which suggests that a smaller sample size is sufficient when the sample is specific, the study aim is clearly focused, and the expected quality of dialogue is high ^28^. All participants will have relevant experience and the sample will be relatively homogenous, thus ensuring high sample specificity. The narrow aim to evaluate the dashboard usability and the think-aloud method employed are expected to yield high-quality, in-depth data. Participants will be individually recruited and consented. If more than 12 volunteer a diverse sample from within this will be selected.

### Data collection

The lead author (CMC) will conduct think aloud exercises and semi-structured interviews. She is a practicing GP who developed the dashboards. She has an existing professional relationship with GPs from one of the recruited sites but otherwise has no existing relationship with potential participants. Participating GPs will be aware of her professional background and of the fact that she developed the dashboards. Microsoft Teams, will be used to screen and audio record the think aloud exercise and subsequent semi-structured interview. Cameras will be turned off during the session, and no video recordings or facial data will be collected. Only screen activity and audio will be recorded for analysis. Recordings will be transcribed using the live transcription feature, and verified manually. During the think aloud exercise GPs will navigate the dashboards and verbalise their thoughts and reactions in real-time to assess how GPs engage with and interpret their data using these visualisation tools. GPs will be reassured that their individual prescribing data will not be stored or reported and that the purpose of the activity is to assess the usability, layout, and clarity of the dashboards. Screen recordings will be deleted after analysis. The GPs will be given four discrete tasks for the think aloud process. This will be followed by the semi-structured interview, where the topic guide will include questions exploring perspectives on prescribing safety, receiving prescribing feedback and views and opinions of the dashboards, see extended data. This approach will provide insights into the usability of the dashboards and how prescribers interpret and act on the feedback provided. The interview topic guide was developed with multidisciplinary input and iteratively refined based on pilot testing. The estimated duration of the entire interview including the think aloud process is 45 minutes.

### Analysis

Transcripts will be read and re-read to support familiarisation with the data and to capture the context and nuances of participants’ responses (CMC). For the think aloud process, screen recordings will be reviewed alongside the transcripts, with verbal data checked and amended for accuracy. Notations will be added to indicate relevant on-screen activity (e.g. navigation paths, pauses, points of confusion), enabling interpretation of how participants interacted with the dashboard in real time. Coding for the think aloud exercises will focus on understanding GPs' engagement with the dashboards, identifying usability issues, and assessing the interpretation and perceived usefulness of the feedback. This will be done using a deductive coding framework based on Nielsen’s five quality components of usability: learnability, efficiency, memorability, error recovery and satisfaction ^29^. These components will form the primary themes, with sub-themes developed from the data. This combined analysis will allow for a richer understanding of usability, layout, and interpretability, by linking verbal feedback with observed behaviour.

An inductive thematic approach will be used to explore GPs’ perspectives on prescribing safety and feedback. The analysis will be organised around three broad topic areas: general views on prescribing safety, experiences and perceptions of feedback, and views on the dashboards. Within each area, themes will be developed inductively from the data, allowing patterns to be identified based on participants’ own words and reflections. This approach will allow themes to be developed directly from the data, helping to capture GPs’ ideas and experiences based on the work of Braun and Clarke ^30^. The transcripts will be systematically coded, with initial coding identifying key phrases, concepts, and categories that are developed from the data. These codes will then be sorted into broader themes that capture the main topics and patterns across the interviews.

## Discussion

### Strengths and limitations

The mixed methods approach will strengthen the depth and richness of the findings; with the think-aloud method allowing for the observation of how GPs interact with the dashboards in real-time and the follow-up interview questions probing how GPs reflect on receiving this feedback. The major limitation of this work is the potential for social desirability bias as the lead author and interviewer (CMC) is also a GP and developed the dashboards. This dual role may discourage some participants from expressing criticism or scepticism openly. However, the interviewer’s clinical background and familiarity with the dashboards also enable more informed probing and interpretation of participants’ responses. To mitigate against the risk of biased interpretation of data interviews and codes will be reviewed by a second member of the study group. The interviews will take place within 2–4 weeks of the dashboards being made available. It is anticipated that some GPs will have explored the dashboards, while others may have limited or no exposure. Apart from notifying practices that the dashboards were available, no specific instructions or encouragement to access them were given. Limited exposure prior to the think-aloud exercise may enhance the assessment of usability and clarity. However, this also limits the opportunity for GPs to reflect on or act upon the data, which may reduce the depth of insight gained during the follow-up semi-structured interviews.

### Implications for practice, policy and research

With recent planned enhancements in e-prescribing and the data infrastructure of Irish primary care, this research will provide important insights into how routine data may be harnessed for audit and feedback purposes. Importantly, it will explore the usability of a prototype dashboard and how GPs interact with this form of data-driven support.

## Patient, public and knowledge user perspectives

In March 2023, at the grant proposal stage, the PI (CMC) met with two public and patient involvement (PPI) contributors to discuss the overall research aims of the proposal and how they would be addressed. Their feedback helped shape the initial proposal. The grant’s independent steering committee includes a GP representative, and the PI is also a practicing GP, ensuring that both patient and clinical perspectives are embedded throughout the project.

## Ethical approval

Ethical approval was granted by the Irish College of General Practitioner Research Ethics Committee, 7 ^th^ October 2024, ICGP_REC_2024_ 2502. All participants will give fully informed written consent.

## Data Availability

As this is a study protocol, there is no data associated with this study. The think aloud tasks, interview guide and SQL code used to generate variables for visualisation in QuickSight are available at: https://doi.org/10.5281/zenodo.15545093 ^31^ Data are available under Creative Commons Attribution 4.0 International
