# Supplementary material for: Developing and assessing the usability of a prescribing quality and safety dashboard in Irish general practice: protocol for a qualitative study
Source: HRB Open Res. 2025 Jun 18;8:67. [Version 1] doi: 10.12688/hrbopenres.14181.1 (PMC12381552; doi:10.12688/hrbopenres.14181.1)
Supplement: Peer review history

# Reviewer report: Reviewer response for version 1

**DOI:** 10.21956/hrbopenres.15596.r48144

**Published:** 2025-08-27

**Authors:** Heike Vornhagen

**Affiliations:** 1 University of Galway, Galway, Ireland

This article describes the intended qualitative study of a prescribing safety dashboard for GPs in Ireland. It firstly introduces the issue of prescribing safety, before laying out the methodology. Overall I think this will be an interesting study though I would suggest some minor changes to the protocol, primarily related to readability. First of all, please check spelling (medication related ham :-) ) and some phrases need to be broken up with commas (e.g. *If more than 12 volunteer a diverse sample from within this will be selected* should be *If more than 12 volunteer, a diverse sample from within this will be selected*).

 Please move this sentence as it is confusing in its current location: 'For metrics displayed as prescribing rates, rates were calculated annually using the total practice population, whereby numbers of private patients were calculated by counting individuals who had at least one encounter in the preceding three years.'  I would move this sentence to the explanation given about GMS and private patients as it would follow more logically the first paragraph under 'Setting'.

 I am also confused as, at the beginning, the authors talk about 'a prescribing safety dashboard' (singular) yet later they talk about 'dashboards' (plural). I presume it is a spelling issue but if not please clarify which other dashboards are meant.

 And please refer to PPI  as 'Proton Pump Inhibitors (PPI) when it is first mentioned.

 Lastly, I would suggest for the authors to review visualisation good practices (for example Jon Schwabisch or Claus Wilke) as some of the graphs visible on the screenshots are problematic and could be easily improved.

Is the study design appropriate for the research question?

Yes

Is the rationale for, and objectives of, the study clearly described?

Yes

Are sufficient details of the methods provided to allow replication by others?

Yes

Are the datasets clearly presented in a useable and accessible format?

Not applicable

Reviewer Expertise:

Data Visualisation, Information System Design, Health Design, Data Dashboards

I confirm that I have read this submission and believe that I have an appropriate level of expertise to confirm that it is of an acceptable scientific standard.

# Reviewer report: Reviewer response for version 1

**DOI:** 10.21956/hrbopenres.15596.r48564

**Published:** 2025-08-26

**Authors:** Douglas Steinke

**Affiliations:** 1 The University of Manchester, Manchester, UK

This is a well-written article of qualitative research into the usability of a prescribing quality and safety dashboard that is used by GPs in Ireland. The article describes the dashboard well so that the reader can understand what is displayed and how it is used. The research questions are clear and given in the article. The methods are well suited to answer the aims of the paper. The methods are described in detail and clear. The results are clearly explained and discussed.

 There are a couple of questions that I have as reviewer that could be considered by the authors.  

 -Investigating the prescribing metric of "High-risk NSAID prescribing and ACE/ARB and diuretic", could the authors just state that it was not possible to identify OTC NSAID use from the data, unless it could be identified. This would make it clear that data is only prescribed NSAIDs that are available. Could this be a limitation?

 -Could the authors include a statement that data saturation was obtained in the analysis of transcripts.  There were a lot of interviews done and I am sure that data saturation was obtained, but it would be good to confirm it in writing.

 -Figures 2 and 3 are not readable. It is not easy to read the text in Figure 2 and Figure 3 is incomprehensible in this state. Please consider a different way of producing the figures so that they are readable in the article.

Is the study design appropriate for the research question?

Yes

Is the rationale for, and objectives of, the study clearly described?

Yes

Are sufficient details of the methods provided to allow replication by others?

Yes

Are the datasets clearly presented in a useable and accessible format?

Not applicable

Reviewer Expertise:

Health Services Research, programme evaluation, drug utilisation research,  pharmacoepidemiology.

I confirm that I have read this submission and believe that I have an appropriate level of expertise to confirm that it is of an acceptable scientific standard.

# Reviewer report: Reviewer response for version 1

**DOI:** 10.21956/hrbopenres.15596.r47950

**Published:** 2025-08-09

**Authors:** Mike O'Callaghan

**Affiliations:** 1 University of Limerick, Limerick, Ireland

This is an important study that outlines a method for harnessing some of the great volume of prescribing that occurs in general practice in Ireland daily. The "live demo" ("think aloud process") aspect of the participants talking through the screens that the researchers/software vendor created to analyse prescribing data is particularly interesting.

 2 minor points for the authors to consider...

 1. I feel the introduction and abstract could be a little bit clearer that the data retrieval and development work has already happened, this only became really clear to me in the "Dashboard Development" section of Methods. Perhaps some of the substantive methods section could be moved to introduction to slim down methods and explain the above a bit more clearly?

 2. I'd suggest the authors expand on the Introduction line- "Given that most prescribing occurs in primary care". Open PCRS data shows general practice in 2023 prescribed 95m items on public schemes (GMS 68m, LTI 11m, DPS 16m) alone. I note some more detail is given in Methods-setting section but framing the scale of what happens in general practice is important.

 With these minor points aside, it's excellent to see this kind of work progressing. It addresses longstanding weaknesses in how Irish GPs currently get feedback on a handful of medicines for GMS patients only, opening up feedback across many other important clinical areas and across patient subgroups (including private patients).

 The dashboard in Figures 2 & 3 looks very promising.

 Finally, mention of annual PCS requirements for GPs/doctors to do work analysing their prescribing/practice may increase relevance for working GPs.

Is the study design appropriate for the research question?

Yes

Is the rationale for, and objectives of, the study clearly described?

Yes

Are sufficient details of the methods provided to allow replication by others?

Yes

Are the datasets clearly presented in a useable and accessible format?

Not applicable

Reviewer Expertise:

General practice, health informatics, data visualisation

I confirm that I have read this submission and believe that I have an appropriate level of expertise to confirm that it is of an acceptable scientific standard.
